# Role of Glycation in Type 2 Diabetes Mellitus and Its Prevention through *Nymphaea* Species

**DOI:** 10.1155/2021/7240046

**Published:** 2021-10-27

**Authors:** Nayab Ishrat, Hamda Khan, Om P. S. Patel, Abbas Ali Mahdi, Farina Mujeeb, Saheem Ahmad

**Affiliations:** ^1^Department of Biochemistry, King George Medical University, Lucknow 226026, India; ^2^Department of Biosciences, Integral University, Lucknow 226026, India; ^3^Centre of Excellence for Pharmaceutical Sciences, North-West University, Private Bag X6001, Potchefstroom 2520, South Africa; ^4^Department of Clinical Laboratory Sciences, College of Applied Medical Sciences University of Hail, Saudi Arabia

## Abstract

The dysregulation of glucose metabolism that includes the modification of biomolecules with the help of glycation reaction results in the formation of advanced glycation end products (AGEs). The formation of AGEs may activate receptors for advanced glycation end products which induce intracellular signaling, ultimately enhancing oxidative stress, a well-known contributor to type 2 diabetes mellitus. In addition, AGEs are possible therapeutic targets for the treatment of type 2 diabetes mellitus and its complications. This review article highlights the antioxidant, anti-inflammatory, and antidiabetic properties of the *Nymphaea* species, and the screening of such aquatic plants for antiglycation activity may provide a safer alternative to the adverse effects related to glucotoxicity. Since oxidation and glycation are relatively similar to each other, therefore, there is a possibility that the *Nymphaea* species may also have antiglycating properties because of its powerful antioxidant properties. Herbal products and their derivatives are the preeminent resources showing prominent medicinal properties for most of the chronic diseases including type 2 diabetes mellitus. Among these, the *Nymphaea* species has also shown elevated activity in scavenging free radicals. This species has a load of phytochemical constituents which shows various therapeutic and nutritional value including anti-inflammatory and antioxidant profiles. To the best of our knowledge, this is the first article highlighting the possibility of an antiglycation value of the *Nymphaea* species by inhibiting AGEs in mediation of type 2 diabetes mellitus. We hope that in the next few years, the clinical and therapeutic potential may be explored and highlight a better perspective on the *Nymphaea* species in the inhibition of AGEs and its associated diseases such as type 2 diabetes mellitus.

## 1. Introduction

Diabetes mellitus causes tremendous social and economic burdens globally. The International Diabetes Federation (IDF) in 2017 revealed a global estimation of 451 million people with diabetes mellitus that is expected to rise to around 700 million by 2045; approximately 5 million deaths are also presently attributable to the disease [1]. It causes a wide range of health complications, which affects almost every organ of the body. The severity of diabetes leads to vascular, retinal, renal, hepatic, and neurological complications that include structural and functional alterations [2]. These accelerate the complications of various pathophysiological mechanisms, including metabolic syndromes and oxidative stress. The primary features of metabolic disorder include weight gain, hyperglycemia, hypertension, cholesterol abnormalities, hyperuricemia, and insulin resistance [3]. Persistent hyperglycemia-associated diabetes plays a crucial role in the development and progression of diabetes-associated secondary complications by facilitating the nonenzymatic protein glycation and accumulation of advanced glycation end products (AGEs) [4]. The hyperglycemic load promotes nonenzymatic covalent reactions between the aldehyde form of sugars with a reactive amino group of lysine and arginine residues of proteins and lipoproteins [5]. This is further rearranged into more stable structures called Amadori products, which undergo oxidation, generating dicarbonyl compounds to form cross-linked structures termed as AGEs [6]. It is much more enhanced in the presence of chronic hyperglycemia due to an increase in glucose. Over time, mitochondrial respiratory chain proteins become increasingly glycated and mitochondrial DNA damage occurs leading to a self-perpetuating cycle of AGE formation and oxidative stress independently of hyperglycemia [7]. Under the influence of oxidative stress, reducing sugars, amino acids, and lipids undergo autooxidation to generate additional reactive carbonyl compounds and increase production of AGEs leading to tissue accumulation [8]. The receptors for advanced glycation end products (RAGEs) with the binding of its different ligands like AGEs, HMGB1, and S100 activate the signaling arrays. The activation of a downstream signaling pathway ultimately leads to the pathophysiological conditions of diabetes, aging, neurological disorders, and cancer, as well as a result of the activation of transcription factors which is responsible for the advancement of many health complications [9]. In an advanced stage, it adversely affects the cellular metabolism, alters the structure and functional properties of proteins, and is involved in various human disorders [8]. In summary, this shows that an extreme AGE level plays a primary role in promoting the initiation and progression of diabetic state-induced complications that include atherosclerosis, nephropathy, and retinopathy [10].

Due to the deleterious effects associated with AGEs, it is important to identify interventions with the ability to prevent AGE formation or inhibit their activity. Several synthetic molecules have been recognized with potent AGE inhibitory activity, although the clinical data on these molecules are still unpublished. These include aminoguanidine and thiazolium salts. Therefore, there is considerable interest in the search for plant-derived molecules with antiglycation activity for the management of diverse diseases. Herbal plants contain secondary metabolites like alkaloids, glycosides, terpene, steroids, flavonoids, and tannins. Polyphenols exhibiting antioxidant activity leads to many health benefits [11]. The *Nymphaea* species (Nymphaeaceae) is the most potent aquatic plant consumed as food and recognized in the traditional system of medicine for the treatment of various life-threatening diseases. The *Nymphaea* species include a wide range of flowering plants. These are called water lilies and are mostly distributed in the tropical areas around the world, living on the banks of lakes and rivers [12]. The plants have a broad range of flower colors, including white, yellow, red, and blue [13]. It is emerging as a new preventive medicine having so many therapeutic properties including antidiabetic activity [14]; hepatoprotective activity [15]; antihyperglycemic and antidyslipidemic activities against T2DM [16]; antiproliferative activity [17]; tumor inhibitory activity; cholinergic activity; analgesic, anti-inflammatory, and antimicrobial activities [18]; and antioxidant activity. There is associated interest within the compounds with antiglycation activity as these will provide therapeutic potential in delaying or preventing the onset of the complications of T2DM. Although several compounds are under study, only a few have successfully moved to the clinical trials but none of these have yet been approved for clinical use.

## 2. Glycation and Its Role in Type 2 Diabetes Mellitus

**Scheme 1:**
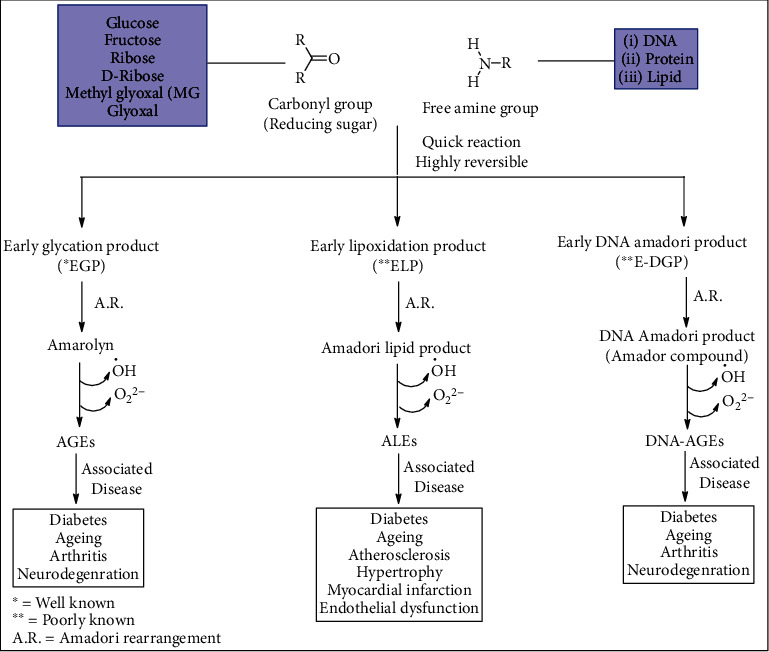
Schematic representation of a probable pathway for macromolecules reacting with reducing sugars to form AGEs/ALEs and DNA-AGEs, respectively. This diagram was adapted from our published research paper in the Elsevier journal “International Journal of Biological Macromolecules” (2013), 58: 206–210. The permission is automatically granted to the authors/corresponding authors of the paper as per Elsevier STM Permission Guidelines (2012).

Glycation is a process of formation of sugar-protein adducts comprised of both reversible and irreversible reactions along with the formation of free radical species. It is a nonenzymatic reaction consisting of an intermediate and end product. The intermediate product is a reversible product known as Amadori product [19] (Scheme 1) or early glycation products, while the end products are irreversible products, such AGEs [9]. Since AGEs are known to be involved in age-associated diseases, such as neurodegenerative and cardiovascular diseases, therefore the age-associated diseases and diabetic complications have been subjects of interest for several years. Moreover, the past investigation from our research team establishes the link between glycation biology with lung cancer [20]. In addition, glycation of apolipoproteins accelerates the development of glycemic condition by producing hemoglobin A1c in people with T2DM. As a consequence, hyperlipidemic condition accelerated foam cell formation. Similarly, glycation of high-density lipoprotein (HDL) involved in oxidative damage by an increase in the generation of free radicals ultimately causes impaired reverse cholesterol transport [21]. On the other hand, it has been reported that AGEs increase reactive oxygen species (ROS) formation and impair antioxidant systems ultimately involved in chronic stress condition in T2DM [22]. The formation of AGEs and their precursors are contributing factors in diabetic complications as per literature survey [23]. T2DM is a complex metabolic disorder that is characterized by insulin resistance or *β* cell dysfunction or both leading to elevated blood glucose levels [24]. Hyperglycemia is suggested to be one of the main causes of T2DM, and it is well known that glycation reaction accelerates the hyperglycemic condition. Similarly, an individual suffering from high blood sugar or age-associated diseases has a high risk of producing AGEs that can build up across the tissue. An enhanced oxidative or carbonyl stress is involved in more rapid and intense accumulation of AGEs in human tissues during aging or age-associated diseases [25]. Apart from the metabolic pathway, the exogenous pathway for the formation of AGEs is dietary AGEs, ultraviolet (UV) irradiation, and smoking habits. The dietary AGEs are formed from the Maillard or browning reactions during cooking involving high dry heat temperatures, such as fried or processed food. The oxidative damage from UV or smoking promotes AGE accumulation along with a large production of free radicals, associated with toxic environmental agents [26]. Therefore, many health experts are suggesting that AGE level is an indicator or biomarker for overall health. The major health problems of the 21st century are age-associated diseases, chronic inflammatory diseases, and other cardiovascular diseases, and these diseases are hallmarked by chronic inflammation *via* oxidative stress and AGEs. The AGE axis and its receptor RAGE are not only linked to alteration in the signaling pathway but also in various age-associated diseases. Moreover, the circulating levels of the soluble form of RAGE (sRAGE) and AGEs are candidate biomarkers for many age-related inflammatory diseases [27] Therefore, to counterattack the risk of diabetes and its complications, it is necessary to target adducts of glycation reaction in every way possible in order to establish an effective therapy.

## 3. Role of Natural Products in the Inhibition of AGEs

**Figure 1:**
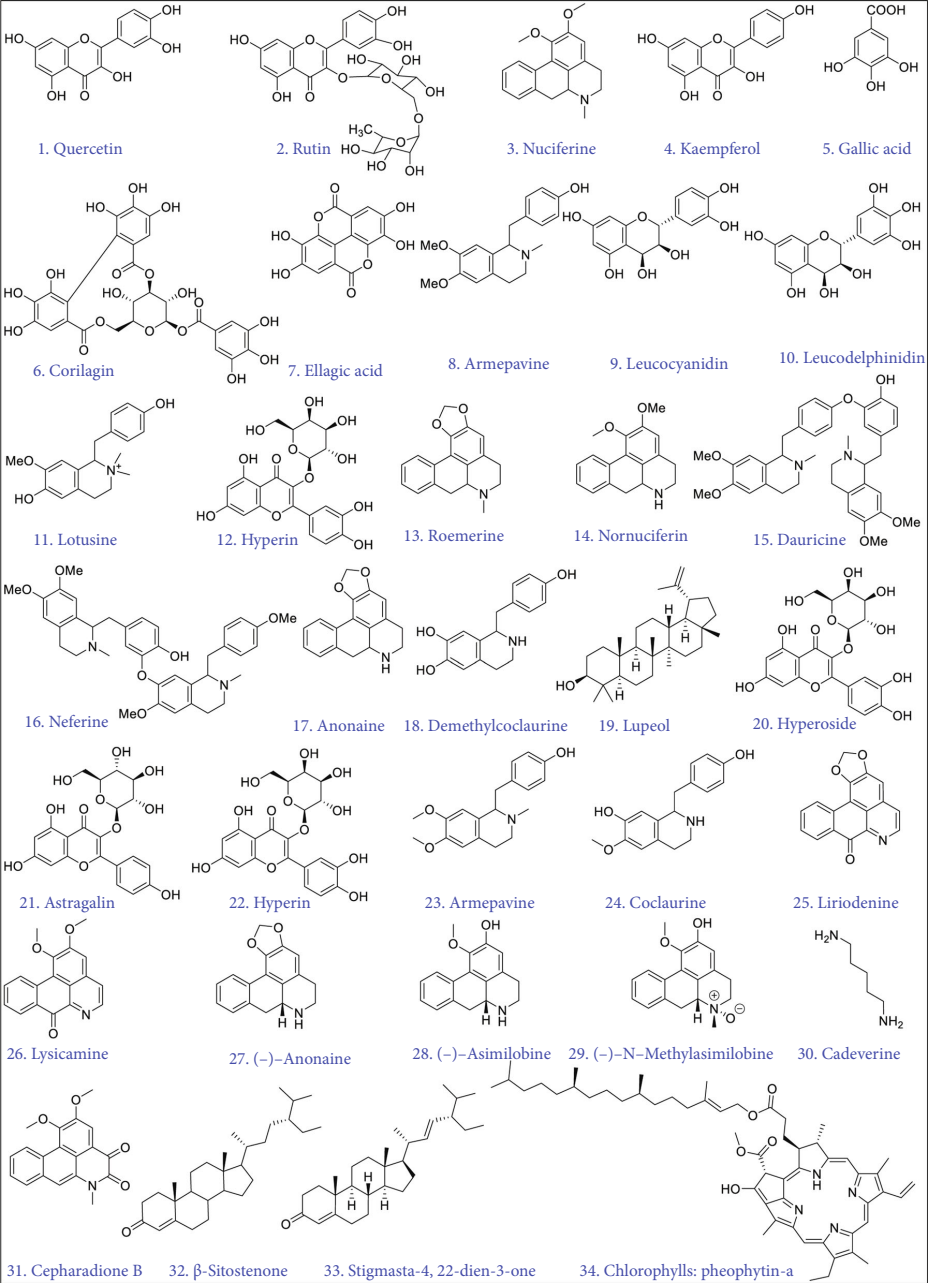
Chemical constituents of the *Nymphaea* species.

An AGE inhibitor such as aminoguanidine is known to inhibit the progression or acceleration of many diseases such as nephropathy, retinopathy, and cardiovascular diseases. Besides, several natural herbal plants are recently proven as AGE-breakers by the way of breaking the cross-linking among the sugar/dicarbonyl compounds including DNA, lipid, and protein. The natural herbs contain a tremendous amount of medicinal compounds with loads of anti-inflammatory properties, antiglycation properties, and antioxidative properties. These natural herbs are easily taken up in the diet as well, for example, *Curcuma longa*, *Nigella sativa*, citric acid, vitamin E, vitamin B_12_, and vitamin C. Additionally, many bioactive compounds and natural inhibitors are known to prevent age-associated diseases such as diabetes mellitus, cardiovascular diseases, and Alzheimer's diseases with the help of the presence of naturally occurring phenolic compounds [28]. From Figure 1, it is very clear that traditional natural products act against the irregularities of signaling pathways in the liver, adipose tissues, muscle tissues, pancreas, and the GI tract. These signaling pathways include PPAR-*γ*, PPAR-*α* and *δ*, PPAR-*γ*, and adiponectin by lowering TNF-*α*, P13K and P38-MAPK, GLUT4, insulin synthesis and secretion cellular signaling, regeneration of *β*-cell, *α*-glucosidase, and *α*-amylase. These natural products act against glycogen synthesis, gluconeogenesis, fat intake, fatty acid synthesis, energy expenditure, insulin sensitivity, glucose uptake, insulin level, and glucose absorption. This management of herbal medicine against metabolic disorder reduces the risk of age-associated diseases and AGE complications.

## 4. *Nymphaea* Species as a New Inhibitor against AGEs

### 4.1. Brief Overview

The genus name *Nymphaea* is adapted from Greek words. Water lily seeds and tubers are consumed as food since few years past by Europeans, Asians, and Africans throughout emergency time. The Egyptians perpetually admire and eat genus *Nymphaea nouchali* and *Nymphaea lotus*. The rootstalk, flowers, and leaves are consumed by the Egyptians, whereas the buds are often delineated on ancient monuments, furnishings, and murals. Water lilies are used in ceremonies throughout Egyptian civilization. Moreover, the Egyptian people believe that the attractive blooms of the water lily portray purity and immortality during the early eighteenth century, and the South African voters consume the rootstock of the blue hydrophytic plant either raw or in curry dishes. Its species *Nymphaea nouchali* has been cultivated in Southeast Asia for centuries, especially around temples. It is additionally cultivated in Sri Lanka and gathered from dried ponds in Asian countries for the rhizomes that are used as food and animal fodder to replace the supply of starch. In Ayurvedic medication, it is customarily used to treat dyspepsia. The species from the Nymphaeaceae family is the most captivating aquatic plants, consumed as food and used as traditional medicine for the treatment of various life-threatening diseases. Another species *Nymphaea pubescens* (Nymphaeaceae) is a long-lived aquatic herb with rootstock, rooting in the mud. Its leaves are long-stalked and leathered, floating on the surface of water, ovate to nearly circular, conspicuously toothed, slightly peltate, twelve to fifteen centimeters across, with a deep bottom. Petioles are long, slender, and submerged. Flowers are fragrant, white or red, about 8 cm in diameter, and borne on long peduncles, while petals are linear-oblong to lanceolate. The fruits are globular, with longitudinally numerous, striated seeds. It is widely distributed in India especially in the winter season. Globally, different parts of the plant species belonging to *Nymphaea* are consumed as food [29].

### 4.2. Photochemistry

The different categories of phytomolecules like alkaloids, glycosides, flavonoid glycosides, hydrolysable tannins, lignans, phytosterols, and triterpenes are present within the numerous species of *Nymphaea* [30]. The isolation and purification of compounds from the plants of the Nymphaeaceae family are extensively studied. The phytochemical constituents of the Nymphaeaceae family have been well established (Table 1) [31, 32]. The presence of two lignans in *N. odorata* [13]; one hydrolysable tannin from *N. tetragona* [33]; several glycosyl flavonols from *N.*×*marliacea* [34], *N. caerulea* [35], *N. lotus* [36], and *N. odorata* [13]; various acylated anthocyanins from *N. candida* [37], *N.*×*marliaceam* [34], and *N. caerulea* [35]; and two rare macrocyclic flavonoids from *N. Lotus* [36] have been reported. Nupharin and nymphaeine have been reported from the flowers of *Nymphaea alba* [38]. Two phenolic base alkaloids of coclaurine have been reported from the aerial parts of *Nymphaea stellatanb* [39]. The cardiac glycoside nymphalin has been reported from the alcoholic flower extract of *Nymphaea alba* [38]. Flavonoids such as anthocyanins, flavonols, and flavones have been reported and presented as flavonoid glycoside with various glycone moieties among the various species of the genus *Nymphaea*. The presence of dimonoside, galactoside, and galactopyranoside attached with delphinidin has been reported from the blue flowers of *Nymphaea gigantean* [40], the leaves of *Nymphaea candida* [37], and the red flowers of *Nymphaea marliaceae* var. Escarboucle [33]. The presence of galactoside and galactopyrano is attached with cyanidin and has been reported from the leaves of *Nymphaea candid* [37] and the red flowers of *Nymphaea marliaceae*, while the presence of rhamnoside, galactopyranoside, and glucoside is attached with myricetin and has been reported from the blue flowers of *Nymphaea caerulea* [33], the alcoholic flower extract of *Nymphaea caerulea* [41], and the ethanolic extract from the leaves of *Nymphaea odorata* [13] and *Nymphaea lotus*. 1,2,3,4,6-Pentagalloyl glucose has been reported from the methanolic extract of the leaves of *Nymphaea lotus* [36]. Methyl gallate has been reported from the methanolic extract of the whole plant of *Nymphaea ampla* [42]. Geraniin, an antimicrobial hydrolysable tannin against fish pathogenic bacteria, has been reported from the acetone fraction of *Nymphaea tetragonna* leaves [33]. *β*-Sitosterol has been reported from the alcoholic flower extract from *Nymphaea alba* [42], while nymphyol has been reported from *Nymphae astellate* [18]. Nymphasterol has been reported from the seeds of *Nymphaea stellata* [43], and *β*-sitosterol and *β*-sitosterol-3-o-*β*-D-glucopyranoside have been reported from the methanolic extract of whole plants of *Nymphaea pulchella* and *Nymphaea gracilli* [44]. Nymphaeoside A and Icariside E4 have been reported from the ethanolic extract of the leaves of *Nymphaea odorata* [13]. Henceforth, the list of valuable compounds from the extraction of the plants from the Nymphaeaceae family has been minutely explained to uncover the role of genus *Nymphaea* in the medicinal field. *Nymphaea* is historically used in Chinese folk drugs to disperse the summer heat and has shown varied health advantages and medicinal activities, as well as inhibitor, medication, antiviral, antiobesity, antiangiogenic, hepatoprotective, immunomodulatory, and hypoglycemic activities. The chemical constituents of *Nymphaea* species are obtained from different plant organs that show many therapeutic properties.

### 4.3. Antioxidant and Anti-Inflammatory Activity of *Nymphaea* Species and Its Relation to Glycation Inhibitory Effect

Free radicals are highly reactive single electron species responsible for the deleterious effect on the human body. Their implications in many diseases like neurodegenerative diseases, cardiovascular diseases, cancer, and T2DM are well established. Several reports have been published on medicinal plants with antioxidant properties such as *Pistacia lentiscus*, *Origanum syriacum*, *Allium sativum*, and *Diospyros abyssinica* [41]. In this section, we have focused on the antioxidant property of *Nymphaea* species, since it is a rich source of phytochemicals and has already been reported as an important medicinal plant used in traditional medicine. The *Nymphaea* species acts as a hepatoprotective agent by inhibiting the effect of free radicals as reported in an *in vitro* study [45]. This evidence provides the use of the *Nymphaea* species against hepatitis. The preventive effect of the *Nymphaea* species was shown in the *in vitro* immunological liver injury of rat primary hepatocyte cultures [12]. Another species of the *Nymphaea* species, that is, *Nymphaea nouchali*, has reportedly shown various biological activities such as anti-inflammatory, antioxidant, antidiabetic, and antihepatotoxic. The seeds of this genus reportedly have an antidiabetic property. *Nymphaea nouchali* belongs to the family Nymphaeaceae and has both antidiabetic and anti-inflammatory properties. The leaves of this plant also possess aphrodisiac and antimicrobial properties. It inhibits the lipid peroxidation activity and also possesses the scavenging activity of nitric oxide [46].

#### 4.3.1. *In Vitro* Antioxidant Activity

*Nymphaea nouchali* leaves were reported to have the presence of compounds like carbohydrates, phenolic compounds, alkaloids, and tannins. In DPPH radical scavenging activity, it showed 94% and 88% of metal chelating activity with IC_50_ values of 42 *μ*g/mL and 28 *μ*g/mL. In its ethanolic and chloroform extract, in spite of having different flavanoid contents, both extracts possess strong antioxidant activity [47]. *Nymphaea nouchali* stem (NNSE) extract also prevented *tert*-butyl hydroperoxide- (t-BHP-) stimulated oxidative stress in RAW264.7 cells by inducing the endogenous antioxidant system and the levels of heme oxygenase-1 (HO-1) by upregulating Nrf2 through the modulation of mitogen-activated protein kinases (MAPK), such as phosphorylated p38 and c-Jun N terminal kinase [48]. The antioxidant activity observed may be due to the presence of tannins and phenolic compounds. In one study, it was found that when Caco-2 cells were treated with 10 mM H_2_O_2_ in combination with the methanol extract of the lotus leaf (0.1–0.3 mg/mL) extract, a dose-dependent protective effect was observed against reactive oxygen species- (ROS-) induced cytotoxicity [49]. This showed new insights into the antioxidant potential and mechanisms of the stem extract of *Nymphaea nouchali* against oxidative stress, which may be a useful remedy for oxidative stress-induced disorders [50].

### 4.4. Anti-Inflammatory Property of *Nymphaea*

Inflammation is a process which induces pathological conditions such as rheumatoid arthritis, cancer, and diabetes. The naturally occurring herbs and the secondary metabolites of the herbal medicine has been used as an anti-inflammatory agent from many years. *Nymphaea* is considered one of the successful anti-inflammatory herbs.

#### 4.4.1. *In Vitro* and *In Vivo* Anti-Inflammatory Activity

The anti-inflammatory activity of ethanolic extracts of *Nymphaea alba* flowers was investigated using acetic acid-induced vascular permeability chronic models in Swiss albino mice [51]. The chloroform fraction of ethanolic extracts of *Nymphaea rubra* flowers were reported to increase the GLUT4-mediated glucose transport and insulin signaling cascades in TNF-*α*-induced insulin resistance in the rat skeletal muscle cells (L6 myotubes) and also decrease Ser307 phosphorylation of IRS-1 by the inhibition of c-Jun NH2-terminal kinase and nuclear factor-*κ* [52] supporting its inflammatory property. In a study, it was reported that *Nymphaea lotus* L. (*N. lotus*) showed anti-inflammatory activity at a conc. of 100–250–500 mg/kg, with diclofenac and hydrocortisone (as positive controls) by paw oedema and skin prick tests in carrageenan-induced Sprague-Dawley rats. It was found that paw oedema sizes decrease in a dose-dependent manner thus supporting that it might contain some anti-inflammatory compounds causing the inhibition of the prostaglandin-induced inflammatory pathway [53]. The *N. hybrida* ethanol extract (NHE) inhibits the LPS-associated inflammatory response by blocking the activation of NF-*κ*B and MAPK pathways in RAW264.7 cells and effectively alleviating the inflammatory response of acute inflammation [54]. *Nymphaea alba* flowers have been reported to have steroids and tannins and possess anti-inflammatory activities. They inhibit the lipid peroxidation activity and possesses the scavenging activity of nitric oxide. Similarly, we know that inflammation is a process that induces pathological conditions such as rheumatoid arthritis, cancer, and diabetes. Additionally, the naturally occurring herbs and the secondary metabolites of herbal medicine have been used as anti-inflammatory agents for many years. Moreover, *Nymphaea* is considered one among the successful anti-inflammatory herbs. The previous in vitro and in vivo data showed the successful anti-inflammatory properties of the ethanolic extract of *Nymphaea*. The polyphenolic extract of *Nymphaea* species have anti-inflammatory properties [55]. However, there is a gap of research in the reference of published data on *Nymphaea* and its role in the inhibition of inflammation and free radicals. In the future, we must consider *Nymphaea* as an attractive model for the inhibition of AGEs and its complications including T2DM.

### 4.5. *Nymphaea* as an Inhibitor for AGEs

*Nymphaea* being a potent inhibitor of free radicals might also act as a powerful antiglycating agent. It has been reported earlier that AGE inhibitors such as pyridoxamine and aminoguanidine significantly inhibit the development of retinopathy and neuropathy in streptozotocin-induced diabetic rats [56]. Treatment with AGE inhibitors is believed to be a possible strategy for preventing lifestyle-related diseases like diabetic complications and coronary artery disease [57]. Most of the species belonging to the family of Nymphaeaceae show antioxidant, antidiabetic, and anti-inflammatory activities. Since the glycation reaction is triggered in the presence of oxidants and/or free radicals [58], it is thus being hypothesized that the Nymphaeaceae family has potential antiglycation activity through the quenching of the free radicals. It is well known that the free radicals which also enhance the formation of AGEs have an impact on the malfunctioning of the metabolic activities. In this way, finding a pathway of *Nymphaea* inhibiting AGEs might act as a therapeutic target in the future.

## 5. Possible Metabolic Syndrome through AGEs and Its Inhibition through *Nymphaea*

Metabolic syndrome (MetS) is a cluster of metabolic abnormalities, raising the risk of patients developing T2DM and cardiovascular diseases [59]. It is a very well-known major cause of death worldwide. A collective study shows that some natural products or molecules can modulate metabolic syndrome (MetS), and the risk factors of MetS are defined as the coexistence of risk factors originating from metabolic origin (insulin resistance, hyperinsulinemia, impaired glucose tolerance, T2DM, visceral obesity, atherogenic, dyslipidemia, and elevated blood pressure) that increases the chances of cardiovascular disease [59]. The major components of MetS are interconnected with each other: (1) Obesity and lack of physical activity increases the level of insulin resistance that is responsible for elevating the level of triglycerides and low-density lipoprotein along with a reduction of high-density lipoprotein [60, 61]. It also favors the growth of atherosclerotic plaques, which ultimately leads to coronary heart and cerebrovascular disease [62, 63]. (2) Insulin resistance contributes to higher levels of serum insulin and glucose precursors in the progression of T2DM [64]. (3) Elevated insulin level leads to excessive renal sodium retention and elevations in blood pressure [65]. (4) Impaired intrinsic cellular expression of endothelial factors leads to increased blood pressure, leading to endothelial dysfunction and decreased nitric oxide production [66].

**Figure 2:**
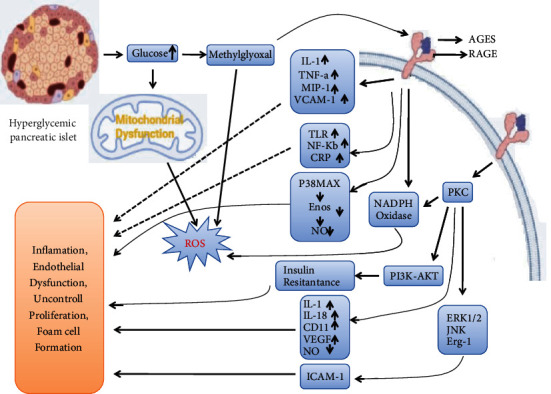
Pathways showing the mechanism of diabetes, oxidative stress, inflammation, and AGEs in metabolic disorder.

In the reference of genetic susceptibility, there are important environmental factors that can influence the pathogenesis of MetS. Positive lifestyle changes can beneficially affect most of the features of MetS [67]. Environmental, genetic, and other metabolic factors are interrelated to the development of T2DM. The advancement of T2DM proceeds with the dysregulation of glucose tolerance and the gradual development of the MetS [68]. MetS is highly rampant in developed countries, affecting around 24% of adults in the United States between the age of 20 to 70 years [69]. The syndrome is also termed as “Syndrome X,” and can be defined as group of abnormalities that includes central obesity, impaired glucose tolerance, T2DM, atherogenic dyslipidemia, hypertension, and coagulopathy [70]. A correlation of diabetes, oxidative stress, inflammation, and production of AGEs that all comes under MetS have been shown in Figure 2, which also increases the risk of cardiovascular disease [71]. We are highlighting a few of the diseases that can occur as a result of AGEs and its harmful effects and supporting the property of *Nymphaea* species against inhibition of these diseases. The deleterious effect of AGE production during progression of metabolic syndrome and its possible inhibition through *Nymphaea* species are follows.

### 5.1. Diabetic Dyslipidemia and Obesity

**Figure 3:**
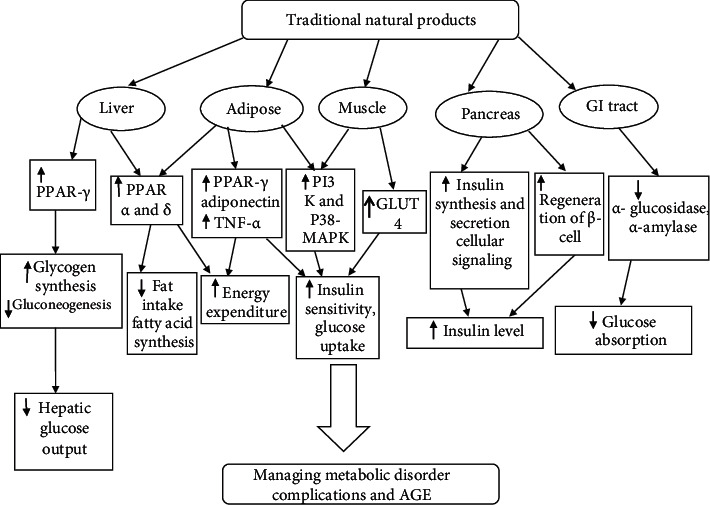
Management of metabolic disorder by natural compounds.

Dyslipidemia is related with T2DM and is observed by cholesterol-rich remnant lipoprotein, very small low-density lipoprotein, moderate hyper-triglyceridemia, and low concentrations of high-density lipoprotein [72]. The composition of lipid particles and gradual increase of low-density lipoprotein cholesterol increases the atherogenicity and the subsequent onset of cardiovascular disease in T2DM patients. Many patients with improved type 1 diabetes may have lipid blood levels that are related with more favorable total cholesterol [73], low-density lipoprotein, and high-density lipoprotein levels that are also normally found in many nondiabetic individuals [72]. Obesity plays a major role in the development of insulin resistance and abnormalities associated with T2DM [73]. Thus, the key strategies recommended for obese patients along with T2DM should adopt the appropriate dietary modifications, routine physical exercise, and loss in body weight to delay the onset of hyperglycemia and dyslipidemia [74, 75]. However, pharmacological interventions are needed in more severe cases of dyslipidemia and hyperglycemia [76]. *Nymphaea* with its different chemical property is responsible for inhibiting the damages through hyperglycemic condition, oxidative stress, and inflammatory condition [77]. Therefore, the prominent factor in obesity is the permanent increase of plasma free fatty acid and the maximum utilization of lipids by muscles that induces reduction of glucose uptake and, subsequently, insulin resistance. An insulin-resistant condition is the key mark to MetS, contributing the major risk factor for the development of T2DM. The hyperinsulinemia condition also supports the mechanism that responds to increased levels of circulating glucose. Individuals who develop T2DM usually show excessive adipogenesis, nuclear peroxisome proliferator-activated receptor modulation, insulin resistance, hyperinsulinemia, pancreatic *β*-cell stress, progressive reduction of insulin secretion, impaired glucose postprandial, and fasting glucose levels [78], and the phytoconstituents present in the *Nymphaea* species so far are reported to control the damage *via* an impaired glucose mechanism (Figure 3). Fasting glucose levels remain normal as long as insulin hypersecretion can compensate for insulin resistance. The decline in insulin secretion causes hyperglycemia that occurs as a late experience and separates the patients with MetS from those with or without T2DM. Therefore, the prevalence of diabetic dyslipidemia and obesity must be in control to avoid the lethal combinations of diseases.

### 5.2. Diabetic Retinopathy

The progression of AGEs and their consequences play role in the development of diabetic retinopathy due to the degradation of several lens proteins and retinal cells [79]. When AGEs bind to their receptor RAGE in the lens, they stimulate the activation of those signaling pathways that are susceptible to oxidative stress and release many local hormones, cytokines, and adhesion molecules [80]. The AGE-RAGE interaction causes apoptosis of vascular cells and thereby result in the death of cells, which is an early sign of retinopathy. The deposited AGEs in the retinal cells stimulate the secretion of interleukin 6 (IL-6), which further induces angiogenesis by increasing the expression of vascular endothelial growth factor (VEGF). Thus, AGEs may cause microvascular changes in the eye of diabetic patients. Additionally, as we discussed earlier, the *Nymphaea* species is reported to be loaded with chemical constituents having anti-inflammatory and antiapoptotic properties which have the potential to heal this; therefore, we can say that the *Nymphaea* species can act as a preventive medicine and act against the damage in the lens protein and retinal cells.

### 5.3. Diabetic Cataract

Glycation reaction in the lens protein of the eyes is a major contributor in the progression of diabetic cataract in later stages. The AGEs generated with lens protein causes a conformational change in the protein, decreasing protein-protein interactions and protein-water interaction. Thus, the transparency of the eye lens is reduced [81]. Experimental studies revealed that an increased AGE formation around the cataract lens is associated with the change of color and opacity of the eye lens [82]. To date, there is not much information available on diabetic cataract in mediation of *Nymphaea.* However, one of the study has been shown that AGE-protein cross-linking can be prevented through natural occurring herbs [83]; therefore, we believe that *Nymphaea* will also prevent the AGE-protein cross-linking the patients with diabetic cataract.

### 5.4. Diabetic Nephropathy

The pathological studies between hyperglycemia and the development of diabetic nephropathy have been responsible for the formation of AGEs. These AGEs and their intermediate species form cross-links with collagen, which cause structural and functional changes in the kidney. AGEs also promote the production of inflammatory cytokines, chemokines, adhesive molecules [84], and growth factors that might be involved in the pathogenesis of diabetic nephropathy. *In vitro* and *in vivo* studies confirmed that the AGE-RAGE axis causes overproduction of matrix proteins which inhibit its breakdown and promote the activation of an oxidative stress pathway that is responsible for damage to organ level [85]. On the other hand, the *Nymphaea* species has been previously mentioned as having an antinephropathic effect, but there is still a research gap in the development of accurate systematic studies in detail as phytomedicine. Henceforth, there is an hour of demand for more research on *Nymphaea* against the AGE consequences.

### 5.5. Neurodegenerative Diseases

The amount of AGEs accumulated in the brain has been noted to increase with the advancement of age. Recent data suggested that the collected AGEs in the brain and other organs of the central nervous system might be responsible for neurodegenerative disorders like Alzheimer's, Parkinson's, proteinopathies, and lateral amylotrophic sclerosis. The amount of AGEs deposited varies according to the pathology of the diseases. A most interesting fact about AGEs is their accumulation at the target organ and also along the path of the nerves. It is now proven that those organs which never form AGE-related compounds are more susceptible to its accumulation. AGEs that are derived by MG are reported to present in diet and that is also capable of penetrating the blood-brain barrier, also enter the glial cells, and can increase the synthesis of amyloids which are responsible for plaque formation [86]. In reference to the role of *Nymphaea* in neurodegenerative diseases, we could not find any relevant article, though the formation of AGEs and neurodegenerative diseases might indicate that there might a possible role of *Nymphaea* in neurodegenerative diseases via inhibition of AGEs.

### 5.6. Cardiovascular Diseases

Accumulated AGEs have been reportedly found during diabetic and nondiabetic conditions. Some of the conditions like smoking, consuming excess deep fried and fatty foods, and food cooked at high temperatures could lead to an increase in AGE concentrations in blood. The deleterious effect of AGEs and their related products are due to their cross-linking nature with matrix proteins, which decreases the flexibility and ultimately causes the dysregulation of the protein. When AGEs are attached with the collagen, elastin, and laminin in the myocardium, it leads to the rigidity and diastolic dysfunction of the heart. Similarly, formation of the AGE-RAGE axis in the myocardium induces fibrosis by enhancing its property of transformation growth factor *β* (TGF-*β*) [87]. AGEs also cause reduction in calcium concentration by slowing the process of the calcium reuptake. AGEs may also delay repolarization of cardiac contractions by decreasing myocardial contractility, and can increase the systolic blood pressure along with endothelial dysfunction. Previously, it was reported that the AGE-soluble RAGE complex cross-links with a low-density lipoprotein, resulting in the decreased uptake by the low-density lipoprotein receptor and its clearance further decreases by macrophage; thus, low-density lipoprotein uptake ultimately leads to atherosclerosis [88]. Thus, AGE accumulation increases left ventricle, end diastolic pressure, diastolic dysfunction, pulmonary congestion, dyspnoea, and systolic heart failure [89]. Additionally, the studies have been done on the role of *Nymphaea* against cardiovascular diseases (Table 1).

### 5.7. Chronic Kidney Diseases

The relationship of the kidney with AGEs could be at its filtration process. The AGEs and related peptides and adducts (the AGE compound is linked to a single amino acid) are filtered by the kidneys, and due to their cross-linking nature, they can bind with epithelial cells of proximal convoluted tubules and then degraded by the lysosomal system, and can further exit through urine [90]. An increased number of AGEs cannot enter into Bowman's capsule and, hence, combine with receptors of the endothelial matrix and form RAGE. The AGE-RAGE axis stimulates the production of various cytokines like the tumor necrosis factor *β* (TNF *β*), activates inflammatory response, and results in glomerulosclerosis. The AGE-RAGE axis also activates NAD(P)H oxidase that further activates oxidative stress and causes decreased capacity of excretion. AGE accumulation in kidneys may cause carbonyl stress, uremia, and loss of its function. In this context, again the significance of the *Nymphaea* species for having active phytoconstituents has been demonstrated and can be treated as a preventive medicine in chronic kidney diseases as well as in cardiovascular diseases.

## 6. Future Perspectives

**Figure 4:**
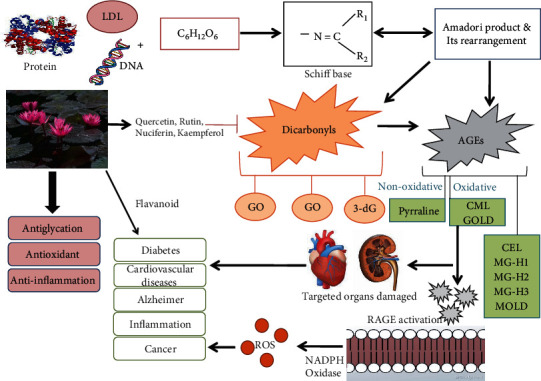
Pathways for AGE formation and its link with different diseases and inhibition by phytoconstituents of the *Nymphaea* species.

Natural products are considered the safest for consumption as compared to other medicinal treatments or clinical candidates derived from synthetic compounds. Over the past few years, some plant extracts, fractions, and compounds were evaluated to check their activities that inhibit AGE formation. From reports that oxidative stress accompanies and accelerates the formation of AGEs, antioxidant compounds [9, 58, 91, 92] therefore appeared as promising agents for the prevention of AGE formation. Previous studies demonstrated that the numerous natural products/compounds act as effective supplements against glycation and are therefore employed in the management and prevention of long-term complications of diabetes mellitus and other related diseases, which is summarized systematically in this article. Despite its valuable significance, there is still a high need to explore their clinical significance, proper physiological attention of inhibitors, and their mode of action to validate their beneficial role as an accompanying treatment in diabetes. Naturally occurring compounds like polyphenols are predisposed by various factors with bioaccessibility, molecular structures, transporters, metabolizing enzymes, etc. and none of the commercially available drugs are free from toxic effect. Therefore, it is extremely important to design theranostic products by using the latest techniques, such as nanotechnology and homogenization, that can improve the bioavailability of natural inhibitors including polyphenols [93], vitamins, and herbal plant extracts, so that we can find a treatment technique with less toxicity. We require polyherbal medications for obtaining the appropriate response towards the nonvulnerable treatment options, since synthetic drugs have adverse effects as usual, and MetS and diabetic complications are multifactorial. However, there is a gap in scientific evidence of plant-derived therapeutic benefits that remains unclear. This review is involved in emphasizing the role of natural products against AGE formation, especially the members from the Nymphaeaceae family. This botanical family is loaded with beneficial chemical properties naturally (Figure 4), with more than 35 different chemical constituents. Its chemical constituents improve the condition of oxidative stress, lower the level of cytokines, and inhibit inflammatory pathways for many years. Many researchers showed its importance in the prevalence of the diseases for many years (Table 1). We conclude that the *Nymphaea* species are the most effective plants with valuable secondary metabolites, and their cellular mechanism might work against glycation, as it is a well-known antioxidant. Also, the *Nymphaea* species and its different plant parts may have more space to further explore the valuable chemical constituents against various metabolic disorders and communicable diseases that has no or the least toxic nature or adverse effects.

## Figures and Tables

**Table 1:** Photochemical composition from eight representative members of *Nymphaea*.

S. no.	*Nymphaea* spp.	Natural product	Reference
1	*N. alba*	Cyanidin 3-(6^″^-acetylgalactoside); delphinidin 3-(2^″^-galloyl-6^″^-acetylgalactoside); delphinidin 3-(6^″^-acetylgalactoside); cyanidin 3-(2^″^-galloyl-6^″^- acetylgalactoside); delphinidin 3-(2^″^-galloylgalactoside; delphinidin 3-galactoside; cyanidin 3-galactoside	Torgil et al., 2001 [41]

2	*N. ampla*	7,3′4′-Trihydroxy-5-O-*β*-D-(2^″^-acetyl)-xylopyranosylisoflavone; 7,3′,4′-trihydroxy-5-O-*α*-L-rhamnopyranosylisoflavone; quercetin 3-rhamnoside; quercetin 3-xylopyranoside; quercetin 3-glucopyranoside; methyl gallate	Marquina et al., 2009 [45]

3	*N. elegans*	Quercetin 3-rhamnoside; *β*-sitosterol; *β*-sitosteryl-3-O-*β*-D-glucopyranoside	Marquina et al., 2009 [45]

4	*N. gracilis*	Methyl 3-O-*β*-D-glucopyranosyloleanolate, 28-O-*β*-D-glucopyranosyloleanolate; 28-O-*β*-D-glucopyranosyl-oleanolate; *β*-sitosterol; *β*-sitosteryl-3-O-*β*-D-glucopyranoside	Marquina et al., 2005 [43]

5	*N. lotus*	Myricitrin; 1,2,3,4,6-pentagalloyl-D-glucose; nympholide A; nympholide B; myricetin-3′-O-(6^″^-p-coumaroyl)glucoside	Elegami et al., 2003 [36], Mukherjee et al., 2009 [12]

6	*N. pulchella*	7,3′4′-Trihydroxy-5-O-*β*-D-(2^″^-acetyl)-xylopyranosylisoflavone; 7,3′,4′-trihydroxy-5-O-*α*-L-rhamnopyranosylisoflavone; kaempferol 3 rhamnopyranoside; *β*-sitosterol; *β*-sitosteryl-3-O-*β*-D-glucopyranoside	Marquina et al., 2005 [43]

7	*N.*×*marliacea*	Myricetin 3-O-(*α*-L-rhamnopyranosyl(1⟶6)*β*-D-galactopyranoside); delphinidin 3-(2^″^-galloyl-6^″^-acetylgalactoside); delphinidin 3-(6^″^-acetylgalactoside); delphinidin 3-galactoside	Fossen et al., 1997 [34]

8	*N.*×*marliacea* var. Escarboucle	Delphinidin 3-(2^″^-galloyl-6^″^-acetylgalactoside); delphinidin 3-(6^″^-acetylgalactoside); cyanidin 3-(2^″^-galloyl-6^″^-acetylgalactoside); delphinidin 3-(2^″^-galloylgalactoside); delphinidin 3-galactoside	Fossen et al., 1997 [34]

## Data Availability

The data is available within the manuscript, if any.
